# Recontacting in clinical practice: an investigation of the views of healthcare professionals and clinical scientists in the United Kingdom

**DOI:** 10.1038/ejhg.2016.188

**Published:** 2017-01-04

**Authors:** Daniele Carrieri, Sandi Dheensa, Shane Doheny, Angus J Clarke, Peter D Turnpenny, Anneke M Lucassen, Susan E Kelly

**Affiliations:** 1 Egenis, University of Exeter, Exeter, UK; 2 Faculty of Medicine, University of Southampton, Southampton, UK; 3 School of Medicine, Cardiff University, Cardiff, UK; 4 Royal, Devon and Exeter Hospital, Exeter, UK

## Abstract

This article explores the views and experiences of healthcare professionals and clinical scientists in genetics about the existence of a duty and/or responsibility to recontact former patients when the genetic information relevant to their health, or that of family members, changes in a potentially important manner. It is based on *N*=30 semi-structured interviews guided by vignettes of recontacting scenarios. The sample included healthcare professionals in the United Kingdom from different medical specialties (clinical genetics, other ‘mainstream' specialties now offering genetic testing), and scientists from regional genetics laboratories. While viewing recontacting as desirable under certain circumstances, most respondents expressed concerns about its feasibility within the current constraints of the National Health Service (NHS). The main barriers identified were insufficient resources (time, staff, and suitable IT infrastructures) and lack of clarity about role boundaries and responsibilities. All of these are further complicated by genetic testing being increasingly offered by mainstream specialties. Reaching a consensus about roles and responsibilities of clinical specialties with regard to recontacting former patients in the light of evolving genetic information, and about what resources and infrastructures would be needed, was generally seen as a pre-requisite to developing guidelines about recontact.

## Introduction

Advances in genomic medicine are producing new interpretations of data that can lead to better diagnosis and treatment of some health conditions. Novel research findings sometimes have implications for previously reported diagnoses and test results. These implications might also be at the level of treatment and surveillance options, such as the identification of PARP inhibitors as a possible treatment for BRCA-related carriers. With the increasing use of whole genome approaches in healthcare, previously discovered variants of unknown significance (VUSs) may now have known disease effects, or they may be re-classified from pathogenic to non-pathogenic. Such reinterpretations are likely to become more common as clinical practice moves away from targeted tests and towards genome-wide approaches, which identify more variants of unknown or uncertain clinical significance. A recent reclassification of BRCA1 c.594-2A>C from pathogenic^1^ has triggered an alert from the UK's Association for Clinical Genetics Science to identify families and individuals who had tested positive for this variant.

Is there a duty or responsibility on the part of clinicians to recontact former patients for whom the interpretation of genetic test results has changed in a clinically important way?

The literature on recontacting is sparse. A recent systematic review by Otten *et al.*^2^ found that a common theme was the clash between the desirability of recontacting and its practical feasibility. The main practical barriers identified were lack of resources (eg, time, staff), and infrastructures (eg, sufficient patient databases).^3, 4^ The main suggestions to help mitigate these barriers were the implementation of digital communication systems between laboratories, clinicians and patients and the involvement of patients and support groups in the process of recontacting – that is, to encourage patients routinely to recontact healthcare professionals (HCPs) for updates.^5, 6^

There is a lack of consistency and clarity around terminology. In the literature, the expression ‘duty to recontact' prevails, but ‘responsibility', ‘obligation' and other terms are sometimes used as synonyms. The systematic review adopts the term ‘duty' and defines it as the ethical and/or legal obligation to recontact former patients in light of new genetic findings.^2^ We follow this definition but we also consider there are important distinctions between ‘duty' and ‘responsibility'.

There is currently no policy, legislation or professional consensus about whether healthcare professionals have a duty to recontact former patients. However, cases where legal liability has been established shed some light on the potential duty to recontact. USA and Canadian courts have established a duty to warn (which is more general than the duty to recontact, and not necessarily directed at a particular patient) in cases of defective drugs or devices. A physician who prescribed treatments, drugs and other medical devices may still have a duty to inform patients if new information about additional risks related to these medical treatments becomes available.^7, 8^ In Tresner v Barke, a California case related to a contraceptive device (Dalkon Shield), a physician who inserted this device has been held liable for not having communicated to patients a later discovery about its side effects.^9^ Focusing completely on the legal interpretation of duty may obscure the ethical complexities involved in recontacting.^10, 11, 12^ For example, to date the courts have considered there is no legal duty of care to relatives.^13, 14^ However, HCPs may still have an ethical duty to contact the relatives of a patient to alert them to actionable risk, as reflected by the recent Australian guidelines which approves direct communication between HCPs and relatives to disclose at risk status in case of a serious threat even without the patient's consent (although the guidelines encourage clinicians to work hard to gain patient consent for disclosure before contacting relatives).^15^

If there is a duty or responsibility to recontact former patients, the next question is with whom would this lie. Genetic testing is beginning to be offered by medical specialists outside clinical genetics, so-called ‘mainstream specialties', for example, cardiology, paediatrics, and oncology; yet these specialties may not receive up-to-date information on DNA variant interpretation. Therefore, the most appropriate lines of responsibility for recontacting could be unclear. Genetic HCPs may be responsible for recontacting patients and families to whom they offer on-going care.^7^ However, much on-going genetic care has devolved to primary and/or secondary care, rather than by genetic services.^16^ For some multisystem disorders, genetic services coordinate care across specialties and this might facilitate appropriate recontact, but coverage to date is uneven. Laboratory staff may learn about reclassifications of VUSs before clinical staff and may be able to notify clinicians,^17^ triggering a recontact.

Other questions include: how long a potential duty or responsibility to recontact might apply, and what types and degrees of information change might justify or trigger recontacting a former patient.^18, 19^ These questions will become more urgent if health care systems move towards the model of universal whole-genome sequencing with the lifetime storage of each citizen's genome sequence to be accessed as required for health care decisions.^20^

There is very limited empirical evidence on recontacting. Our recent survey was the first study to specifically explore current recontacting practices in regional clinical genetics services.^21^ This showed that services in the UK do recontact former patients but in an *ad hoc* fashion. More than half of the services were uncertain about whether formalised recontacting systems were desirable. Some argued that implementing such systems would give patients more choice and lead to a better standard of care. Others expressed concerns that establishing a duty to recontact may create a worrisome legal precedent that would be difficult to enact universally. In order to provide much needed empirical evidence, this paper draws on interviews with healthcare professionals from clinical genetics, professionals from mainstream specialties, and scientists working in genetic service laboratories. It provides an in-depth investigation of their perspectives on the clinical, ethical and legal issues related to recontacting.

## Methods

The interviews we conducted are part of a broader study to investigate ethical, legal and social issues related to recontacting in clinical practice in the NHS in the United Kingdom (study website: http://ex.ac.uk.//mgc). The sample comprised healthcare professionals and laboratory scientists (*n*=30) recruited via NHS trusts and professional societies (see Table 1). Participants were identified and invited to participate by the clinical authors of this paper based on their professional experience and networks. In this sense the sampling strategy was purposive. A multi-site strategy was adopted to gain a sample of participants from different medical specialties. Interviews were semi-structured and face-to-face, except for one conducted via Skype. We investigated: experiences of recontacting; views about situations in which recontact may/may not be regarded as a good standard of care; and views about potential responsibilities for recontacting and the implementation of policies and systems to enable this. The interviews were guided by three vignettes (about new diagnosis, reclassification of a VUS, and new treatment for an old finding). Vignettes are increasingly recognised as useful tools in interviews where the objective is to query responses to variation in clinical practice or to situations posing professional and/or ethical quandaries regarding action, and are useful in obtaining ‘accounts' of both action and reasoning.^22^ We designed the vignettes based on the literature, clinical experience, and survey findings^21^ and piloted them with the first few participants.

Interviews were recorded, transcribed verbatim and subjected to thematic analysis.^23^ The interviews were independently analysed by three members of the research team. Key themes were identified and discussed in regular team meetings. The interview guide and vignettes are provided as Supplementary Information A and B.

## Results

### Sample

The categories below refer to the respondents' main role at the time of the interview. However some of the respondents had more than one clinical area of interest and varied clinical backgrounds. The HCPs and laboratory staff interviewed were mostly very experienced and occupied senior positions. To gain a mix of perspectives (in particular from those who were more recently trained) a small number of less senior HCPs (trainees doctors in medical genetics) was also recruited.

### No standard recontacting practices

In line with the findings from our survey,^21^ the genetic HCPs reported that they recontact patients occasionally. The most frequently mentioned cases were: the availability of new genetic tests or new results; family follow up (eg, a new family member is referred to the clinic and this triggers a review of the family files); reclassifications of VUSs; reproductive relevance (eg, when children of parents with a genetic condition reach reproductive age); recruit former patients to participate in research projects. However, there was no uniform recontacting system; the cases discussed were the result of diverse personal systems they had developed, often reliant on their own memory.

*I've seen thousands of patients in my time here. If there's a new gene, I might remember some of them. Sadly we have no efficient database to recall patients. So when I leave, the memory of those patients is gone (Genetic consultant 1)*

As illustrated, these personal systems were often felt to be inefficient and unsustainable. For some this is likely to be exacerbated in the context of expanding services (as more patients are referred to genetic services, remembering each one becomes less possible). Recontacting appeared to be bespoke to the specific patient and/or family. Deciding whether or not to recontact, and how to do so, often required a review of the patient case and clinical history.

*I think reviewing the individual cases is important because sometimes it's not appropriate to recontact, sometimes that person has passed away and there's no more relevance, sometimes you know that the family structure is such that now would not be a good time… (Genetic counsellor 1)*

Some genetic HCPs expressed concern towards the patients potentially missed due to the current *ad hoc* recontacting practices:

*At the minute it [recontacting] is opportunistic. If I remember the family very well and I remember that they want to be recontacted or if it comes across my desk because of an audit, or another project, or another family member and it's obvious that they wish to be recontacted and there's something to be gained, but there must be many files filed away that I know nothing about where patients could benefit (Genetic consultant 7)*

Databases, or the lack thereof, were mentioned frequently. Indeed, a common theme across all participants was that there was a tension between the desirability in principle of recontacting, and its practical feasibility given the constraints in the NHS.

*I think it would be good to do it. I think it's also one of those many things within the NHS where there is an uncomfortable mismatch between what we would ideally like to do and what we have the capacity to do. So in an ideal world, yes we would recontact. [But] when you have to make a choice between recontacting and serving other clinical needs we may not (Genetic counsellor 1)*

As this participant alluded, HCPs thought recontact important, but had to make careful judgements about how best to allocate their limited resources.

### Unclear lines of responsibility

Another important perceived barrier to recontacting was the lack of clarity about roles, including whose responsibility it would be to initiate any recontact. This was complicated by the increasing use of genetic testing by mainstream medical specialties. The majority of respondents considered genetic HCPs their preferred recontact route – given their ability to communicate complexities of genomic information, and, as the quotation below suggests, because of existing patient registers.

*If we want to do an audit in cardiology I think cardiology can't even identify patients with hypertrophic cardiomyopathy, whereas we can… not perfectly, but we can identify notes and pull them [...]. I think the cardiologists would… they have told me, ‘Why haven't you recontacted them?' (Genetic consultant 7)*

Some genetic HCPs, however, challenged this view, arguing that other specialties more involved in on-going treatment, and the patients themselves, should share responsibility for recontacting, for example if their clinical situation changed or the patient was planning a family, was pregnant, or had a child. According to this view, genetic HCPs' role is not to actively recontact, but rather, it is patients' and other healthcare professionals' role to recontact genetic HCPs to ask for updates.

*They need to recontact us, recontact doesn't work one way. I think it should come from the patients and families. And the doctors who officially are paid to look after their care […] I feel there's less responsibility on us to recontact people as on people and other specialists, doctors, to recontact us to find out what's changed since (Genetic consultant 1)*

There was also lack of clarity between genetic HCPs and clinical scientists in the laboratory in relation to whose role it should be to keep up to date with the reclassification of variants that trigger recontact. Some genetic HCPs expected to be updated by the laboratory about reclassifications.

*I feel that it's more the responsibility of the laboratory who has done the testing to actually notify the clinicians that they* [VUSs] *have been re-classified. I've had a case recently […] there is a VUS that has been identified in the family that is now classified as pathogenic and for me to be able to use it I need the laboratory to re-issue the reports and in this case it's been me who has come back to the laboratory, but I feel it's more their responsibility to notify me (Genetic consultant 3)*

Some clinical scientists argued for a two-way responsibility between the laboratory and HCPs, and highlighted how the laboratory normally responds to genetic HCPs' requests.

*I can't possibly be a specialist in every clinical area. I'm a head of a lab but we provide services for 1800 different disorders. I try to be very responsive to a clinician asking the question because they know their patients, they know those disorders, That's where I see my role and the lab's role is to be responsive to that. But then within the laboratory you also have scientists who're specialists in certain scientific areas and I think they also have a role to bring to the attention of the service, to me and of the clinical team [that] there is this new development, there is a new gene. So I think we've got a responsibility, the responsibility is two-way (Head of laboratory)*

### Recontacting requires multidisciplinary collaboration

Rather than identifying a specific specialty as being responsible for recontacting, others have argued that this responsibility should be shared among all the medical specialties and laboratory scientists involved in the diagnosis, treatment and management of patients.

This suggestion was corroborated by the recontacting cases (both related to the vignettes and HCPs' own practice) discussed during the interviews. For example, decisions made by genetic HCPs about whether and how to recontact often required collaboration with colleagues, mainstream specialities, and the laboratory. Collaborations were also mentioned in relation to the review of the accuracy and clinical significance of new genetic information (eg, VUSs). Multidisciplinary collaborations were regarded as one of the most effective ways to reduce misunderstandings about roles and responsibilities between healthcare professionals in the management of patients.

*The multidisciplinary process, it's the diagnosis, it's the management, it's the information pipelines, it's the wider family issues, and if you are not doing that then you are not addressing the problem at the right level. If you try and fragment it [...] without a doubt things will be missed and they will be missed simply because there's pressure on time, there's pressure on people, etc… …. I think however it's done, in this age of rapidly expanding knowledge, understanding and uncertainty, you've got to have mechanisms that are going to address it (Genetic consultant 4)*

### Patients should (sometimes) share responsibility

Some respondents argued in favour of the idea that patients should share responsibility for recontacting by agreeing to contact healthcare professionals when an event in their family happens that is relevant (eg, a new birth), and at regular intervals to ask for updates. This was presented as being advisable with current limited resources, and in line with the trend to give patients more autonomy and control over their health.^24^

*I always say to patients that as things change we can't guarantee [recontact], so you should recontact us if anything changes in your family, or if you read anything that concerns you and you want to know if it's relevant […] a lot of people are very reactive and not proactive about their health I absolutely think people should recontact […]. I definitely think there's a shared responsibility, it's their health (Genetic consultant 5)*

However, a few healthcare professionals' pointed out that patients are diverse (in terms of their needs, education, socio-cultural backgrounds, and their capacity for autonomy), and so the shared responsibility model will not always be appropriate:

*I think they can share that responsibility, but I think they should have the right for us to take full responsibility. If they choose not to, and I'm thinking of many of the children I see with a neurodisability, their parents may be affected by a similar learning disability, it would be unreasonable to expect them to take some responsibility for that. So I think they have to be allowed to give up that responsibility, and for us to take it (Paediatrician 1)*

### A consensus about recontact is needed

The majority of respondents found the lack of consensus about the existence of a duty to recontact, and the potential professional and legal consequences of (not) recontacting, to be problematic.

*You could be sued in either direction if you are going to worry about those things. I know some people within our team are very conscious of the legal aspects. If you didn't give someone that information […] then there is an implication there. How a court would judge it in terms of how far does your responsibility go, I don't know. I think in a way there may be court cases in the future and I don't feel that I'm clear on… well what's the ground rule, how far are we expected to go, where does our formal legal responsibility end up? (Genetic counsellor 1)*

Some expressed skepticism towards the idea of introducing legislation or guidelines, arguing that these would stifle clinical practice and judgment. However, the need to reach a professional consensus about the existence of a responsibility or duty to recontact was highlighted by the vast majority of respondents.

*I think as a specialty, as a group of health professionals we really need to have an awful lot of considered debate to say actually what do we do with all this information? (Genetic counsellor 2)*

Reaching this consensus – that is, agreeing and clarifying the role(s) of clinical genetics and other specialties – was generally seen as a key pre-requisite to any decision about developing recontacting guidelines and/or legislations. Another important prerequisite was the clarification of necessary resources and infrastructures.

*I think pragmatically this requires a watertight data automation, any guideline or best practice... should dictate how the dataset is kept and monitored. If this is reliant on people recalling, or making an effort, or personal reading, and therefore thinking, ‘I've read article A, and I'll therefore go and seek for this...' that will fail. If it is too ad hoc, it's better in a way not to have it, and so that people know they have to keep checking (Paediatrician 1)*

Finally, the need to have more information about patients' expectations of, and preferences for, recontact, and to address the ‘gap' between the expectations of patients' and healthcare professionals' was also highlighted.

*Do patients believe that if there was something out there we would let them know? Because if they are relying on that, and it's not happening, then that's quite a worrying gap (Genetic counsellor 1)*

## Discussion

Overall our findings support the idea that recontacting is an issue of increasing concern in a health service engaging with the rapidly evolving field of genomic medicine. Our participants thought it to be a desirable standard of care, but were uncertain whether effective guidelines were possible or desirable. The data highlight several issues that now need urgent consideration in order to decide whether and how recontacting should be implemented.

Currently, recontacting is ‘opportunistic', as described by one respondent, and this raises the question of equitable healthcare service provision. More efficient automated patient databases may mitigate some of the resource issues, helping healthcare professionals to identify patients who may benefit from a recontact (or to keep patients up to date if models of shared responsibility are adopted), and allow for more, and more consistent recontacting. However the recontacting that does take place appears to be bespoke to individual patients and families and cognizant of the sensitivity of communicating new information. Complete automation would not allow for this bespoke element of recontacting.

Clarifying professional roles and responsibilities for recontacting – for example, in relation to who (between genetic HCPs and clinical scientists in the laboratory) should keep up to date with the reclassifications of VUSs – is of pressing importance. Because of this lack of clarity, we are hesitant to adopt the term ‘duty' and we prefer the term ‘responsibility' (we noticed that also our respondents tended to use the word ‘responsibility', rather than ‘duty'). The term ‘duty' appears to establish a binding framework of liability which presupposes clarity about who should perform such a duty. ‘Responsibility' can instead be used more broadly to denote the existence of a potential obligation when there is lack of clarity about who is involved in this obligation. Interestingly, the term ‘responsibility' lends itself more easily than ‘duty' to describe situations in which patients may also be involved in recontacting. It makes more sense to say that patients could sometimes share with HCPs the responsibility for recontacting – as some respondents suggested (eg, by agreeing to contact HCPs at regular intervals to check for updates) – rather than share HCPs' duty to recontact. We are planning to investigate this important issue of differences between ‘duty' and ‘responsibility' further. We are aware that this issue is also linked to the ambiguity between legal and professional forms of governance – which is another key concern to healthcare professionals.

Professional debates involving genetic HCPs and other specialties attempting to reach an agreement about whether a duty or responsibility to recontact exists and whose role it would be to recontact may offer a suitable opportunity to start addressing some of these key recontacting issues. We suggest a few questions for consideration to guide future policy and professional debates about whether and how recontacting should take place (see Table 2 below).

We recognise that the problem of recontacting does not arise solely in relation to genetic testing. There have been recent cases of patient recall for example in relation to PIP (Poly Implant Prothese),^25^ and to concerns that a dentist may have transmitted Hepatitis C infection to patients.^26^ Both these examples involved widespread dissemination of information to the public, setting up helplines, and requiring the patient to share some responsibility for contacting healthcare professionals. More evidence, possibly also from other specialties, is needed to inform any debate about whether and how to implement recontacting systems.^2^

The interviews we conducted represent only a phase of a broader ongoing study. We are currently investigating patients' expectations regarding responsibilities and mechanisms for recontacting. We also aim to disseminate our findings to other stakeholders (including patient groups, and relevant professional organisations), and we expect this to lead to a discussion about drafting professional guidance regarding recontacting in the UK (and possibly other countries), or working toward a professional framework, as appropriate.

There are some limitations to this study. Although varied, the sample is limited; other HCPs in the UK may express different views. Moreover, interviews were undertaken in the UK which has a National Health Service and a long-established clinical genetics service; the findings may not be applicable to the rest of Europe and to other countries which have different models of healthcare systems, and/or less established clinical genetics services.

## Figures and Tables

**Table 1:** Participant occupations

Clinical genetics healthcare professionals	Genetic consultants	10
	Genetic counsellors	5
	Trainee doctors in clinical genetics	4
		
Clinical genetics scientists	Scientist	2
	Head of laboratory	1
		
Mainstream healthcare professionals	Paediatricians	3
	Cardiologists	2
	Haematologists	1
	Endocrinologists	1
	Oncologists	1

**Table 2:** Key recontacting questions

**Qualitative questions**
What are the differences and implications, if any, between professional, legal, and ethical duties or responsibilities to recontact?
Whose professional role(s) would it be to recontact?
What does holding information mean in terms of responsibility or duty?

**Practical questions**
In what situations would it be relevant (eg, only when new information has tangible impact on patient management)?
How often, and over what span of time, would genetic services be expected to conduct repeat laboratory analyses or bioinformatic reinterpretations on any samples or results that included VUSs?
Which methodologies and infrastructures, if any, might be useful in recontacting systems?
