# Supplementary material for: Recontacting in clinical practice: an investigation of the views of healthcare professionals and clinical scientists in the United Kingdom
Source: Eur J Hum Genet. 2017 Jan 4;25(3):275–9. doi: 10.1038/ejhg.2016.188 (PMC5315519; doi:10.1038/ejhg.2016.188)
Supplement: Supplementary Information [file ejhg2016188x1.docx]

# SUPPLEMENTARY INFORMATION

1. **Recontact vignettes**

**Diagnosis**

1. A new gene is discovered, which when mutated, is the explanation for a group of previously undiagnosed patients. Each genetic service probably has about 50 patients they have seen over the years who might have this diagnosis. Whilst the clinical team bear this new diagnosis in mind with any new referral, they do not have the time/ funding/IT support to track back through patients seen over the years who might have this diagnosis.

**VUS**

1. Chloe has developmental delay and dysmorphic features. A CGH reveals a small deletion (a Variant of Unknown Significance, VUS) with no obvious genes that would explain her symptoms. Three years later a research publication describes other children with developmental delay and this same deletion- that is the VUS is reclassified as pathogenic.

**Treatment**

1. A new drug that can delay the onset of some cases of Huntington’s disease has been developed (the news received some press coverage). The genetic services in the UK holds records of over 5000 people who might benefit from the drug. Three clinical genetic services decide to contact all individuals, but the other services feel that they do not have sufficient resources and this is better left to the Huntington Disease Association and GPs.
2. **Interview guide**

**Professional experience**

1. Can you just tell us a little about your background?
   1. What are your current roles and responsibilities? For how long have you worked in this role?
   2. Do you order genetic tests in your work?
2. Have you recontacted patients? [specify genetics if necessary]
   1. If yes, go to Q. 3
   2. If no, go to Q. 4
3. [For those who have recontacted] Can you describe how you have recontacted patients?
   1. How do you word a letter/manage a phone that is about recontacting? Do you use some standard words or phrases/a kind of template?
   2. Do you recontact different patients differently?
   3. Why did you recontact that/those patient(s)?
   4. Did you discuss this with your colleagues?
   5. What was the trigger? [the lab reclassified a gene; new understanding in the literature]
   6. How do you manage a recontact through the patient referral system?
   7. Do you recontact even if you can’t offer treatment for the new diagnosis?
   8. How do you ensure you recontact all relevant patients? [OR] To what extent do you rely on memory to identify relevant patients?
4. [For those who have not recontacted] Why not?
   1. Are there circumstances in which you would recontact patients?
   2. If there were systems in place that would support recontacting patients, would you recontact more? [that recontacting could be done consistently for all relevant patients]
   3. Are you aware of colleagues who have recontacted patients? What is your view of these decisions?
5. [For professions not core to genetics] Could you describe your usual practices (or those of your profession) concerning genetic testing?
   1. Who, amongst healthcare professionals involved in a case, has responsibility for a patient’s genetic information?
   2. Do these practices vary within your own speciality (e.g. in your own team, in different centres across the country)? If so, could you give some examples of significant differences?
   3. Are these practices different from those of other healthcare specialists who may be/are involved in re-contacting? How?
6. [For all professionals] Do you make patients aware that they might be recontacted in the future?
   1. If so, how?
   2. Do you record patients’ preferences about whether and how to be re-contacted? How?
   3. If this this is not part of your practice, why is this case?

**Vignettes**

1. [Diagnosis vignette]
   1. Do you think that other stakeholders, e.g. the laboratory, patient advocacy groups or the media, should share responsibility for re-contacting? If so, what role should they play?
2. [VUS vignette]
   1. What do you think your patients’ expect with regarding to being re-contacted?
   2. What type of information do you consider to be significant enough to trigger a re-contact?
   3. Do you need to be able to offer a plan or treatment?
   4. Are there circumstances when you think you might recontact just to inform someone of a genetic diagnosis?
   5. Does your team/service have a shared approach on the matter?
3. [Treatment vignette]
   1. Appropriate standard of care
   2. Would you consider the potential economic costs to the wider NHS of recontacting patients to inform them of a diagnosis?
   3. Role of patient advocacy groups

**Views**

1. Do you think healthcare professionals have a duty/responsibility to re-contact patients if they become aware of new genetic information that may have an impact on their health?
   1. Does this duty extend to other potentially affected family members?
   2. How important is professional judgement in relation to this duty?
   3. Do you think the courts should regulate this duty?
2. If patients indicate they do not want to be re-contacted, are there circumstances where healthcare professionals should re-contact them anyway?

**Responsibilities**

1. What are the lines of responsibility between different healthcare professionals in relation to re-contacting?
2. Do you think that, alongside healthcare professionals, patients should also share responsibility for re-contacting?
   1. If so, how would this model of shared responsibility work?
   2. If not, why – in your opinion –would this not be the case?
   3. How do you think your colleagues within and outside your speciality would answer this question?

**Policy**

1. Do you think clinical genetic services should implement routine re-contacting systems?
   1. If yes, how? Do you think guidelines would be useful?
   2. If no, why not?

1. Is there anything you would like to add?

*Thank you very much for your time*
